# Chronic granulomatous disease presenting as retinal mass

**DOI:** 10.1186/1757-1626-1-257

**Published:** 2008-10-21

**Authors:** Ahmad M Mansour, Mays Al Dairy, Rola Hamam, Ahmed A Hidayat

**Affiliations:** 1 Department of Ophthalmology, American University of Beirut, POB 113-6044, Beirut, Lebanon; 2 Division of Ophthalmology, Armed Forces Institute of Pathology, Washington, DC, USA

## Abstract

1-year-old girl was admitted for fever of unknown origin. Funduscopy revealed juxtapapillary retinal inflammatory mass in one eye with a differential diagnosis of sarcoidosis, tuberculosis, retinoblastoma or metastatic disease. Retinal biopsy showed necrotizing granulomatous retinitis. Extensive workup and therapeutic trials failed to confirm the diagnosis of tuberculosis or sarcoidosis. Her 7-month brother and 4-year-old male cousin presented with nystagmus, poor vision, paravascular pigmentary changes and were initially diagnosed as recessive retinal dystrophy. The girl died at age 2 from tuberculous meningitis and the boys had recurrent tuberculous and Aspergillus infections. Awareness of the typical fundus findings in chronic granulomatous disease allows early diagnosis of the disorder.

## Case presentation

**Figure 1:**
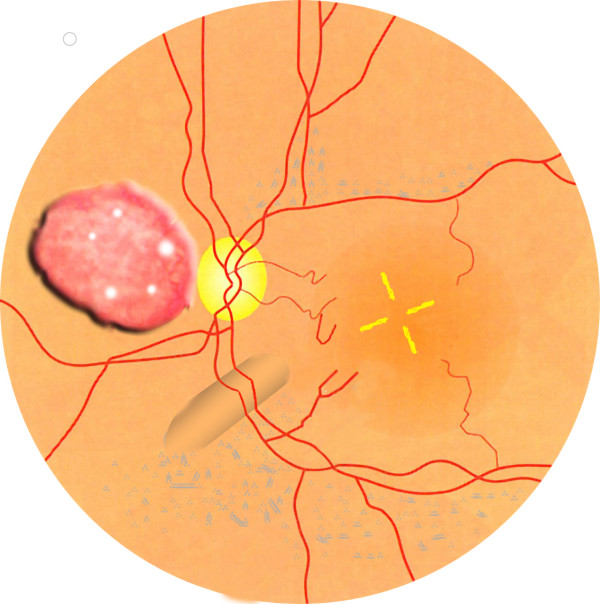
**Funduscopy**. Arstistic depiction of the retinal mass of the left fundus in this 1-year-old girl. The differential diagnosis of this multinodular inflammatory mass with exudative retinal detachment and circinate hard exudates was tuberculosis, sarcoidosis, metastatic tumor, and retinoblastoma.

**Figure 2:**
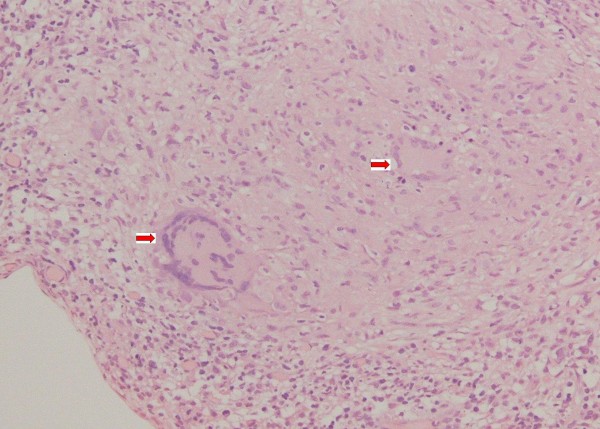
**Retinal biopsy**. Retinal biopsy in this 1-year-old girl shows Touton giant cell with negative Ziel-Nelson, Fite Ferraco, periodic acid Schiff, and Gomori methenamine silver stains.

**Figure 3:**
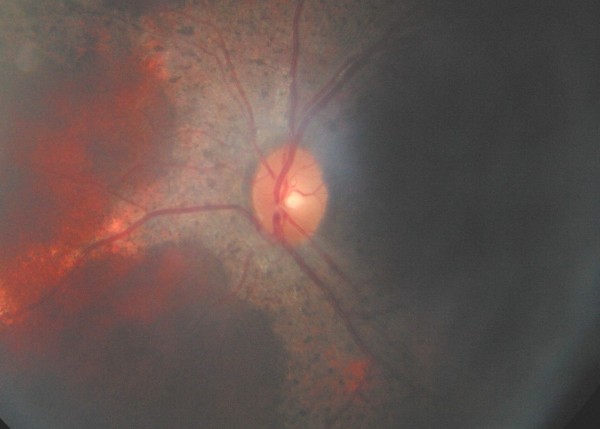
**Brother's fundus**. Diffuse pigmentary loss of the paravascular retinal pigment epithelium in the left fundus of the 7-month old brother with nystagmus.

This 1-year-old girl with high fever (40°C) of unknown origin had a juxtapapillary multinodular 4 disc diameter vascular inflammatory mass with exudative retinal detachment in the left eye (fig. 1). Diagnostic tests and therapeutic trials failed to confirm the diagnosis of tuberculosis or sarcoidosis, with retinal biopsy done to rule out retinoblastoma (fig. 2) showing necrotizing granulomatous disease of unknown etiology (negative stains for mycobacteria). Her 7-month brother and 4-year-old male cousin presented with nystagmus, poor vision, paravascular pigmentary changes (fig. 3) and were diagnosed as recessive retinal dystrophy as the parents were first-degree cousins. The girl died at age 2 from tuberculous meningitis and the boys had recurrent tuberculous and Aspergillus infections.

## Discussion

Chronic granulomatous disease is a syndrome that presents as pneumonia, infectious dermatitis, and recurrent abscess formation beneath the skin and in multiple organs, including the eye [1]. Tissue examination typically shows microscopic granulomas [2]. There is a recent shift in the most common infecting organisms away from staphylococci and enteric bacteria to *Aspergillus *species [1]. At the cell level, neutrophils and macrophages phagocytose but do not kill all organisms effectively as the NADPH oxidase complex does not convert oxygen effectively to microbicidal oxidants.

Around 76% of patients had the X-linked recessive form of chronic granulomatous disease [1]. Chorioretinitis may be more common than previously appreciated (23.7% [3] vs. 35.3% [4]), and boys with the XLR disease should have routine full eye exams. Fig. 3 displays the typical "punched out" chorioretinal lesions with pigment clumping along major retinal vessels (that may interfere with vision) seen in up to one third of patients with chronic granulomatous disease [3,4]. Occasionally the presentation is that of a retinal mass [5] as in the present case. Chronic granulomatous disease needs to be included in the differential of inflammatory retinal mass and the clue lies in family screening for paravascular pigmentary retinopathy.

## Consent

Written informed consent was obtained from the patient's family for publication of this case report and accompanying images. A copy of the written consent is available for review by the Editor-in-Chief of this journal.

## Competing interests

The authors declare that they have no competing interests.

## Authors' contributions

The manuscript has been read and approved by all the authors, that the requirements for authorship in this document have been met, and that each author believes that the manuscript represents honest work. AMM prepared the manuscript. AH confirmed the diagnosis by performing all the histopathologic work. RH and MD assisted in data collection and manuscript preparation.
